# Associations Between Nonspecific Blood-Derived Inflammatory Indices and MRI-Derived Frontal Network Degeneration in Progressive Supranuclear Palsy

**DOI:** 10.3390/neurolint18090161

**Published:** 2026-08-24

**Authors:** Bartosz Migda, Michał Kutyłowski, Natalia Madetko-Alster, Anna Migda, Karol Kutyłowski, Piotr Alster

**Affiliations:** 1 Diagnostic Ultrasound Lab, Department of Pediatric Radiology, Medical University of Warsaw, 02-091 Warsaw, Poland; 2 Department of Radiology, Mazovian Brodno Hospital, 03-242 Warsaw, Poland; michael.kutylowski@gmail.com; 3 Department of Neurology, Medical University of Warsaw, 02-091 Warsaw, Poland; natalia.madetko@wum.edu.pl (N.M.-A.); kutylowski.karol@gmail.com (K.K.); piotr.alster@wum.edu.pl (P.A.); 4 Department of Endocrinology, Diabetology and Internal Medicine, Medical University of Warsaw, 02-091 Warsaw, Poland; anna.migda@wum.edu.pl

**Keywords:** progressive supranuclear palsy, neuroinflammation, magnetic resonance imaging, volumetric analysis, blood-derived inflammatory indices

## Abstract

Background: Progressive supranuclear palsy (PSP) is a primary 4-repeat tauopathy in which neurodegeneration may be accompanied by neuroinflammatory and peripheral immune alterations. Whether peripheral inflammatory activity reflects structural degeneration within vulnerable brain networks remains unclear. Methods: This retrospective case–control study included 12 patients with PSP and 12 patients with Parkinson’s disease (PD). Automated volumetric analysis of 3-Tesla MRI was performed using volBrain 2.0. Blood-derived inflammatory indices included neutrophil-to-lymphocyte ratio (NLR), monocyte-to-lymphocyte ratio (MLR), systemic inflammation response index (SIRI), and red blood cell distribution with coefficient of variation (RDW-CV). Results: Patients with PSP showed significantly lower normalized superior frontal gyrus and pallidal volumes than patients with PD. Within the PSP group, higher values of selected blood-derived inflammatory indices were associated with lower frontal network volumes. After adjustment for age, MLR was inversely associated with the composite Frontal Network Score (partial r = −0.7333, *p* = 0.0067, FDR q = 0.020). The strongest regional association was observed between SIRI and medial frontal cortex volume (rho = −0.748, *p* = 0.0051); however, regional associations did not remain significant after FDR correction. Conclusions: Peripheral inflammatory markers were associated with MRI-derived measures of frontal network degeneration in PSP. The association between MLR and the composite Frontal Network Score supports a link between systemic immune alterations and network-level neurodegeneration in PSP.

## 1. Introduction

Progressive supranuclear palsy (PSP) is a four-repeat tauopathy and clinical syndrome with an incidence rate of around 2.6 per 100,000 person-years [1]. Since its original description in the 1960s, the clinical and pathological concepts of PSP have evolved substantially. PSP as a pathology is associated with the presence of tufted astrocytes. The symptomatological aspects are linked to oculomotor dysfunction, akinesia, postural instability and cognitive/language impairments [2]. The features of the entity have undergone significant extensive analysis throughout the 60-year history of research. The pathological view of the disease is currently assessed in terms of the pathways affecting particular regions of interest. The assessment of the course of degenerative abnormalities enables more insightful evaluations on the causes of gradual clinical deterioration. The analysis of PSP staging performed by Planche et al. showed initiation within the pallido-nigro-luysian system, spreading rostrally through the striatum and the amygdala to the cerebral cortex and caudally to the brainstem [3]. The evolution is currently examined in the context of its association with certain impacting factors. In PSP the pathomechanism remain incompletely understood. Among the possibly relevant features linked to the disease’s background are neuroinflammation and peripheral inflammation. Other properties of the mechanisms are likely related to metabolic abnormalities and environmental factors, among others. The inflammatory background of PSP has not been verified as it is interpreted as a possible cause and consequence of the disease [4]. This, combined with ongoing research concerning neurodegenerative changes, leads to the necessity of evaluating possible links between inflammatory factors and atrophic abnormalities, which could be obtained by performing biochemical and neuroimaging assessments. Despite growing evidence supporting both network-based neurodegeneration and neuroinflammatory involvement in PSP, studies integrating peripheral inflammatory markers with quantitative MRI measures of regional brain atrophy remain scarce.

PSP is clinically heterogeneous and encompasses several phenotypes defined by the predominance of specific clinical manifestations. In addition to the classical Richardson syndrome (PSP-RS), characterized predominantly by early postural instability and vertical ocular motor dysfunction, recognized phenotypes include PSP with predominant parkinsonism (PSP-P), progressive gait freezing (PSP-PGF), corticobasal syndrome (PSP-CBS), predominant speech/language disorder (PSP-SL), and other less common presentations. These phenotypes may differ in their clinical course and regional patterns of neurodegeneration, which is relevant when interpreting neuroimaging findings [2,5].

Evidence supporting immune involvement in PSP has emerged from neuropathological, molecular imaging, and peripheral blood studies. Microglial activation has been demonstrated in vivo using translocator protein (TSPO) PET and has been associated with subsequent clinical progression, while recent post-mortem validation supports the interpretation of TSPO binding as reflecting activated microglial populations [6,7]. Regional neuroinflammatory changes have also been shown to spatially parallel tau pathology across functionally connected regions in primary 4-repeat tauopathies [8]. In addition to innate immune mechanisms, increased cytotoxic T-cell infiltration in the PSP midbrain suggests involvement of adaptive immune responses [9]. Evidence for peripheral immune alterations is more limited, although increased NLR and changes in circulating neutrophil profiles have been reported in PSP compared with healthy controls and, in some studies, PD [10,11,12]. Collectively, these observations support an interaction between immune dysregulation and PSP pathology, while the relationship between indirect blood-derived immune indices and structural neurodegeneration remains insufficiently characterized.

The hematological indices examined in the present study capture partly different components of systemic immune and inflammatory status. NLR reflects the relative balance between circulating neutrophils and lymphocytes, whereas MLR additionally emphasizes the monocyte compartment, which is relevant to innate immune and monocyte–macrophage pathways. SIRI integrates neutrophil, monocyte, and lymphocyte counts into a composite index and may therefore capture a broader shift in circulating immune-cell balance. RDW-CV differs biologically from these leukocyte-derived indices, reflecting variability in erythrocyte size and representing a nonspecific hematological parameter that may be influenced by systemic inflammatory and metabolic processes. Importantly, none of these indices directly measures neuroinflammation or inflammatory activity within the central nervous system [12,13,14,15,16,17]. These indices were selected because they are readily derivable from routine complete blood count data, represent complementary components of peripheral immune and hematological status, and have previously been investigated in neurodegenerative and parkinsonian disorders [10,11,12,13,14,15,16,17]. NLR has specifically been studied in PSP [10,11,12], whereas inclusion of MLR and SIRI extends the assessment to monocyte-related and composite leukocyte responses. RDW-CV was included as a biologically distinct hematological index rather than as a direct measure of immune-cell balance. Their combined evaluation was exploratory and was not intended to establish disease-specific inflammatory biomarkers.

Similar interactions between peripheral and central inflammatory processes have been investigated in other neurodegenerative disorders, particularly Alzheimer’s disease (AD). Altered circulating cytokines and other inflammatory mediators have been repeatedly reported in AD [18], while molecular imaging studies have demonstrated regional associations between microglial activation and tau pathology [19]. Several mechanisms may link systemic immune alterations with central neurodegenerative processes, including circulating inflammatory mediators, blood–brain barrier dysfunction, peripheral–central immune signaling, and modulation of microglial responses [20]. These observations suggest that peripheral inflammatory changes may accompany or modulate neurodegenerative processes, although their causal contribution to structural brain loss remains uncertain.

Recent neuroimaging studies indicate that PSP-related neurodegeneration follows the architecture of interconnected fronto-striato-thalamo-cortical networks rather than affecting isolated brain regions, with particular involvement of structures implicated in executive and behavioral functions [21,22,23,24,25]. At the same time, accumulating evidence from experimental, molecular imaging and neuropathological studies suggests that neuroinflammatory processes may contribute to disease progression [4,6,7,8,9]. Whether peripheral inflammatory activity reflects structural degeneration within these vulnerable networks remains largely unknown.

The regional MRI analysis was therefore focused a priori on structures representing major cortical and subcortical components of the fronto-striato-thalamo-cortical system implicated in PSP. These included the frontal lobe and selected frontal cortical regions, together with the caudate nucleus, pallidum, thalamus, and brainstem. Neuropathological and neuroimaging studies demonstrate progressive involvement of these structures in PSP, while network-based studies indicate that their degeneration reflects disruption of interconnected motor, executive, and behavioral circuits rather than isolated regional atrophy [3,21,22,23,24,25]. Therefore, the aim of the present study was to investigate the relationship between peripheral inflammatory indices and MRI-derived measures of frontal network atrophy in PSP and to compare selected volumetric measures between PSP and Parkinson’s disease.

## 2. Materials and Methods

### 2.1. Study Design and Participants

This retrospective case–control study included patients diagnosed with progressive supranuclear palsy (PSP) and Parkinson’s disease (PD) who underwent brain MRI as part of their routine clinical evaluation at the Department of Neurology, Medical University of Warsaw. Diagnoses were established by movement disorder specialists according to the Movement Disorder Society criteria for PSP and Parkinson’s disease, respectively [2,26]. The study cohort consisted of 12 patients with PSP and 12 patients with PD. Standardized disease-severity measures, including temporally matched UPDRS (Unified Parkinson’s’ Disease Rating Scale) and Hoehn and Yahr scores, were not consistently available for all participants because of the retrospective design and were therefore not included in the comparative analyses. PD was selected as the comparison group because it represents a clinically relevant differential diagnosis of PSP in patients presenting with parkinsonian motor symptoms. The comparison was therefore intended to place the PSP findings within a clinical parkinsonian context rather than to distinguish PSP from other primary tauopathies. Written informed consent was obtained from all participants at the time of enrollment into the original study protocol approved by the Ethics Committee of the Medical University of Warsaw.

Demographic and clinical data were obtained from medical records. Age and sex were recorded for all participants. Patients with major structural brain abnormalities, extensive cerebrovascular disease, intracranial tumors, or MRI examinations of insufficient quality for volumetric analysis were excluded.

### 2.2. Laboratory Assessment

Peripheral inflammatory burden was assessed using routine complete blood count measurements obtained during the clinical diagnostic work-up. All blood samples were analyzed in a single laboratory, the Department of Laboratory Diagnostics, Mazovian Brodno Hospital, Warsaw, Poland, thereby minimizing potential inter-laboratory variability. Hematological measurements were performed using a Sysmex XS-1000i automated hematology analyzer (Sysmex Corporation, Kobe, Japan).

The following inflammatory indices were calculated: neutrophil-to-lymphocyte ratio (NLR), monocyte-to-lymphocyte ratio (MLR), and systemic inflammation response index (SIRI). NLR was calculated as the ratio of neutrophil count to lymphocyte count, MLR as the ratio of monocyte count to lymphocyte count, and SIRI as neutrophil count × monocyte count divided by lymphocyte count. Red blood cell distribution width coefficient of variation (RDW-CV) was obtained directly from complete blood count reports.

### 2.3. MRI Acquisition and Volumetric Analysis

Brain MRI examinations were performed using a 3-Tesla Siemens scanner (Siemens Healthineers, Erlangen, Germany) equipped with a standard head coil at the Department of Diagnostic Imaging, Mazovian Brodno Hospital, Warsaw, Poland. The imaging protocol included a high-resolution three-dimensional T1-weighted Magnetization-Prepared Rapid Gradient-Echo (MPRAGE) sequence with an isotropic voxel size of 1 × 1 × 1 mm. All MRI studies were reviewed by an experienced radiologist to verify image quality and exclude major structural abnormalities that could interfere with volumetric analysis.

Anonymized MPRAGE DICOM datasets were converted to NIfTI format and processed using the volBrain 2.0 online platform. Automated segmentation and volumetric analysis were performed with the vol2Brain pipeline, which provides tissue-specific, cortical, and subcortical volumetric measurements using multi-atlas label fusion and automated error-correction procedures [27].

The analyses were restricted to a predefined set of MRI regions selected on the basis of current neuropathological and neuroimaging evidence in PSP. These regions included the frontal lobe, medial frontal cortex (MFC), superior frontal gyrus (SFG), anterior cingulate gyrus (ACgG), caudate nucleus, pallidum, thalamus, and brainstem. Together, these structures form key components of the fronto-striato-thalamo-cortical network, which is consistently involved in PSP and is closely linked to executive dysfunction, motor impairment, and disease progression [23,24,25]. To limit the number of statistical comparisons, only predefined regions of interest were analyzed. All volumetric measurements were expressed as percentages of intracranial volume.

### 2.4. Statistical Analysis

Statistical analyses were performed using Statistica version 13 (TIBCO Software Inc., Palo Alto, CA, USA). The distribution of continuous variables was assessed with the Shapiro–Wilk test. Since most variables were not normally distributed and the study groups were relatively small, non-parametric methods were used for all analyses.

Continuous variables are presented as medians with interquartile ranges (IQRs), whereas categorical variables are reported as absolute numbers and percentages. Comparisons between patients with progressive supranuclear palsy (PSP) and Parkinson’s disease (PD) were performed using the Mann–Whitney U test for continuous variables and Fisher’s exact test for categorical variables.

Relationships between inflammatory blood markers and MRI volumetric measures were assessed using Spearman’s rank correlation coefficients. Correlation analyses were performed separately for the PSP and PD groups. Because multiple correlations were tested, *p*-values were adjusted using the Benjamini–Hochberg false discovery rate (FDR) procedure. Adjusted q-values below 0.05 were considered statistically significant.

To evaluate the potential influence of age on the observed associations, additional analyses were performed using age-adjusted partial correlation coefficients.

A composite Frontal Network Score was calculated to provide an integrated measure of frontal network involvement. For each participant, normalized volumes of the frontal lobe, medial frontal cortex (MFC), superior frontal gyrus (SFG), and anterior cingulate gyrus (ACgG) were transformed into z-scores using the mean and standard deviation derived from the Parkinson’s disease (PD) group as the reference population. The resulting z-scores were subsequently averaged to obtain a single summary measure of frontal network integrity. Lower Frontal Network Score values indicated greater frontal network degeneration relative to the PD reference group. Associations between inflammatory markers and the Frontal Network Score were analyzed using the same statistical approach as for individual volumetric variables, including age-adjusted analyses and correction for multiple comparisons using the Benjamini–Hochberg false discovery rate procedure.

All statistical tests were two-sided. A *p*-value below 0.05 was considered statistically significant unless otherwise specified.

## 3. Results

### 3.1. Demographic and Inflammatory Characteristics

The PSP and PD groups did not differ significantly with respect to age or sex distribution. NLR, SIRI, and RDW-CV were numerically higher in the PSP group, whereas MLR was slightly lower in PSP. None of the between-group differences in peripheral inflammatory indices reached statistical significance (Table 1).

### 3.2. MRI Volumetric Differences Between PSP and PD

**Figure 1:**
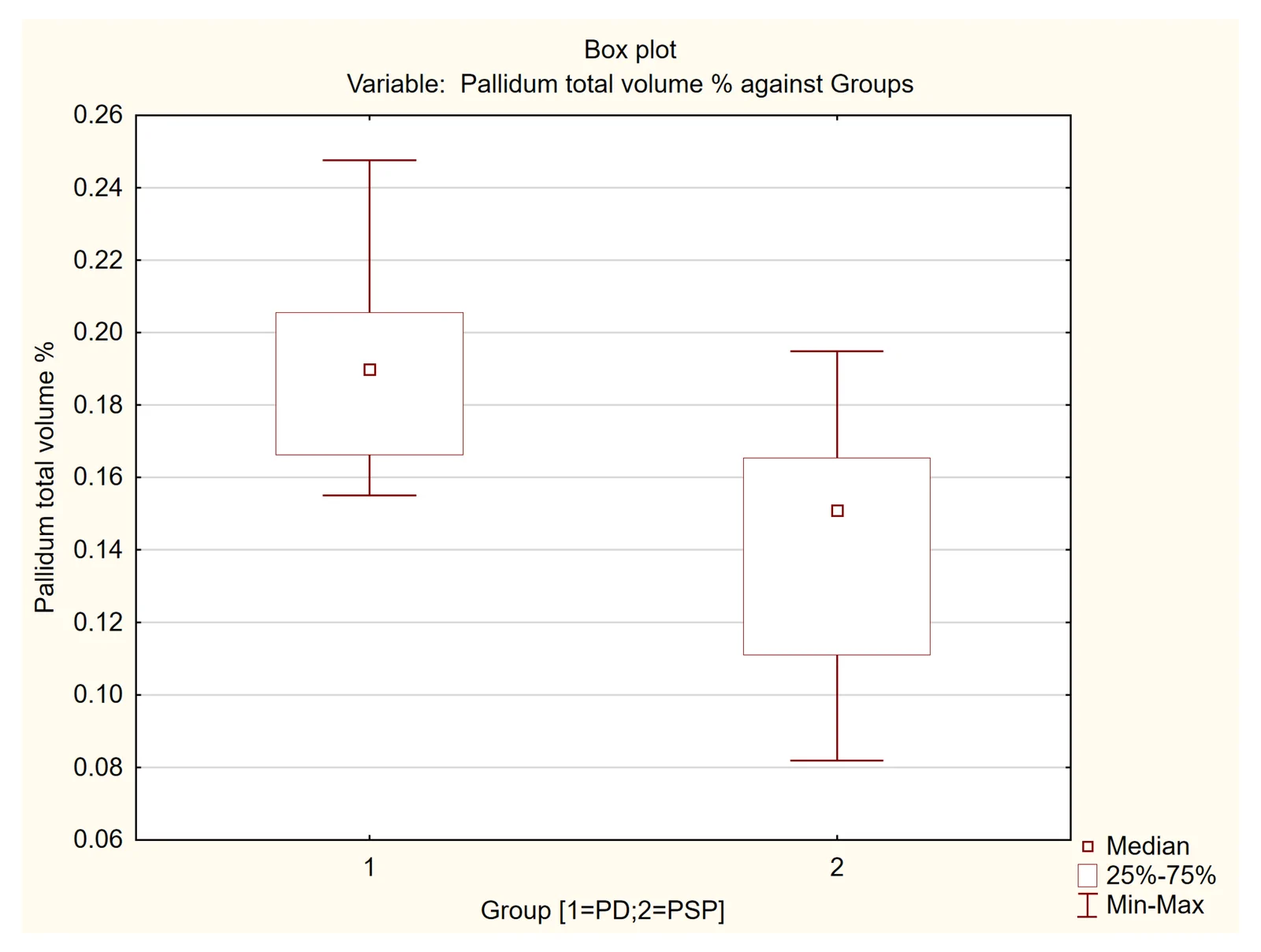
Comparison of normalized total pallidal volume between patients with Parkinson’s disease (PD) and progressive supranuclear palsy (PSP). Box plots show the median, interquartile range, and minimum–maximum values. Patients with PSP demonstrated significantly lower normalized pallidal volume than patients with PD (Mann–Whitney U test, *p* = 0.00165; FDR q = 0.0074).

**Figure 2:**
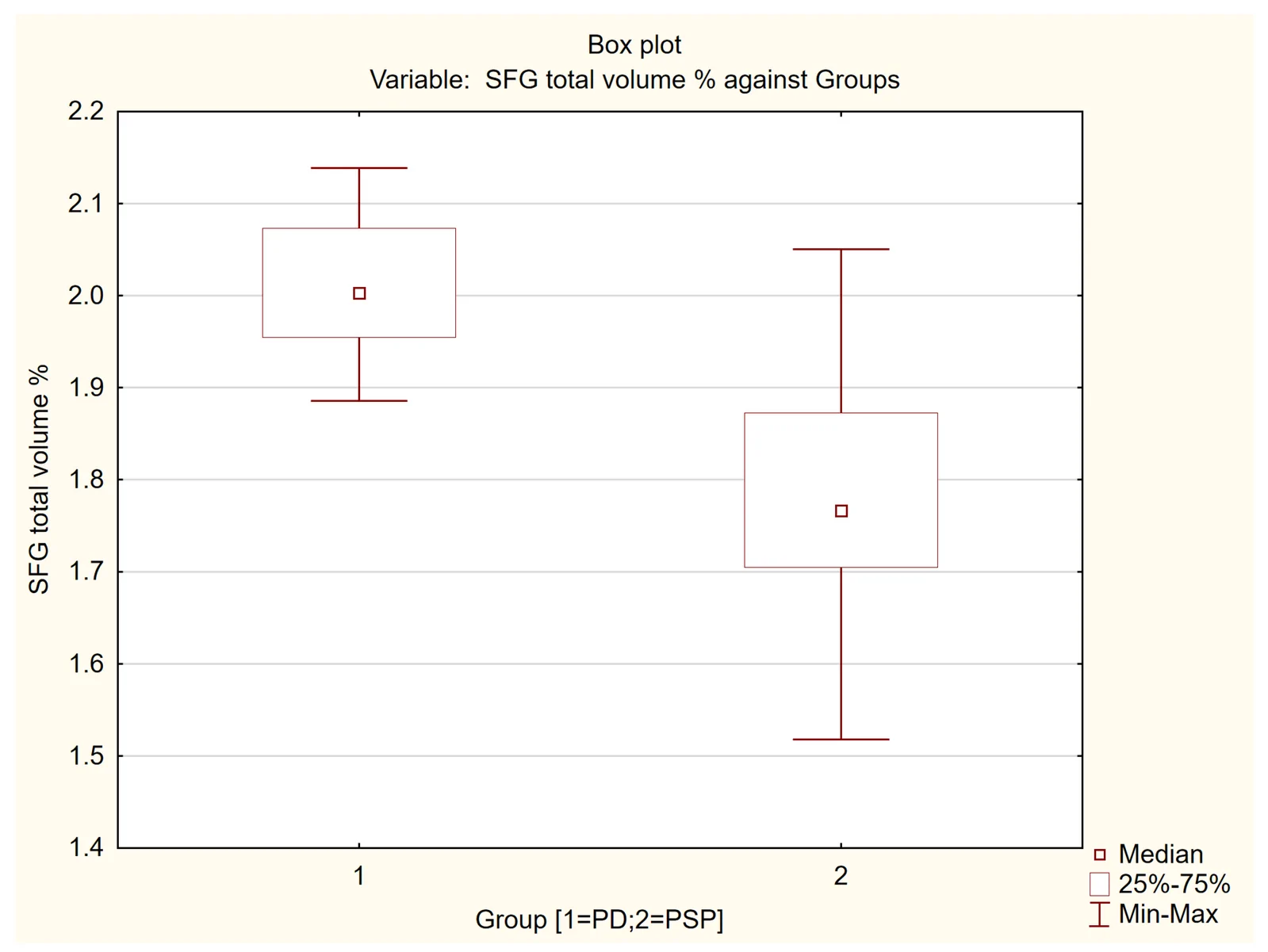
Comparison of normalized total superior frontal gyrus (SFG) volume between patients with Parkinson’s disease (PD) and progressive supranuclear palsy (PSP). Box plots show the median, interquartile range, and minimum–maximum values. Patients with PSP demonstrated significantly lower normalized SFG volume than patients with PD (Mann–Whitney U test, *p* = 0.00048; FDR q = 0.0043).

Among the predefined regions of interest, patients with PSP showed significantly lower normalized volumes of the superior frontal gyrus and pallidum compared with patients with PD. Both findings remained significant after correction for multiple comparisons (Figure 1 and Figure 2, Table 2). Specifically, SFG and pallidal volumes were substantially lower in PSP.

Additional trends toward lower frontal, caudate, and brainstem volumes were observed in PSP, although these associations did not remain significant after FDR correction (Table 2).

### 3.3. Associations Between Inflammatory Markers and Frontal Network Volumes in PSP

**Figure 3:**
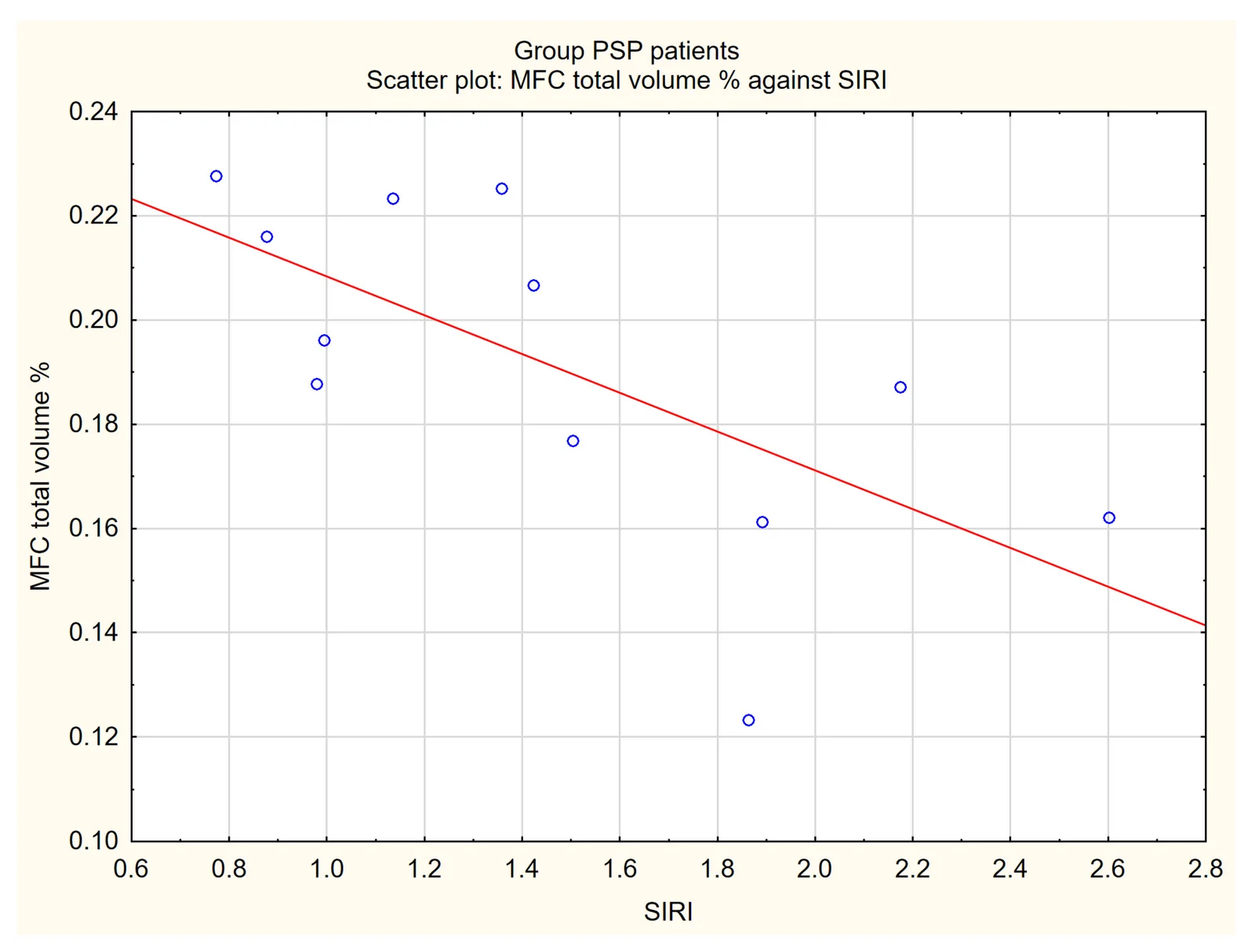
Association between the systemic inflammation response index (SIRI) and normalized medial frontal cortex (MFC) volume in patients with progressive supranuclear palsy (PSP). Each point represents one participant. The red line illustrates the linear trend. Higher SIRI values were associated with lower normalized MFC volume (Spearman’s rho = −0.748, *p* = 0.0051).

Correlation analyses revealed a consistent inverse relationship between peripheral inflammatory burden and frontal network integrity in PSP. The strongest association was observed between SIRI and MFC volume (rho = −0.748, *p* = 0.0051, Figure 3). Similar negative correlations were found for NLR and MLR with both MFC and SFG volumes (Table 3). Although none of the regional correlations remained formally significant after correction for multiple testing, all significant associations demonstrated the same direction of effect, indicating that higher inflammatory marker levels were associated with lower frontal network volumes.

### 3.4. Age-Adjusted Analyses

Adjustment for age did not materially alter the observed relationships. The strongest association remained between SIRI and MFC volume (partial r = −0.734, *p* = 0.0066), while negative correlations between NLR, MLR and frontal network structures persisted with comparable effect sizes (Table 4). These findings suggest that the observed relationships were not primarily driven by age-related changes in brain volume.

### 3.5. Composite Frontal Network Score

**Figure 4:**
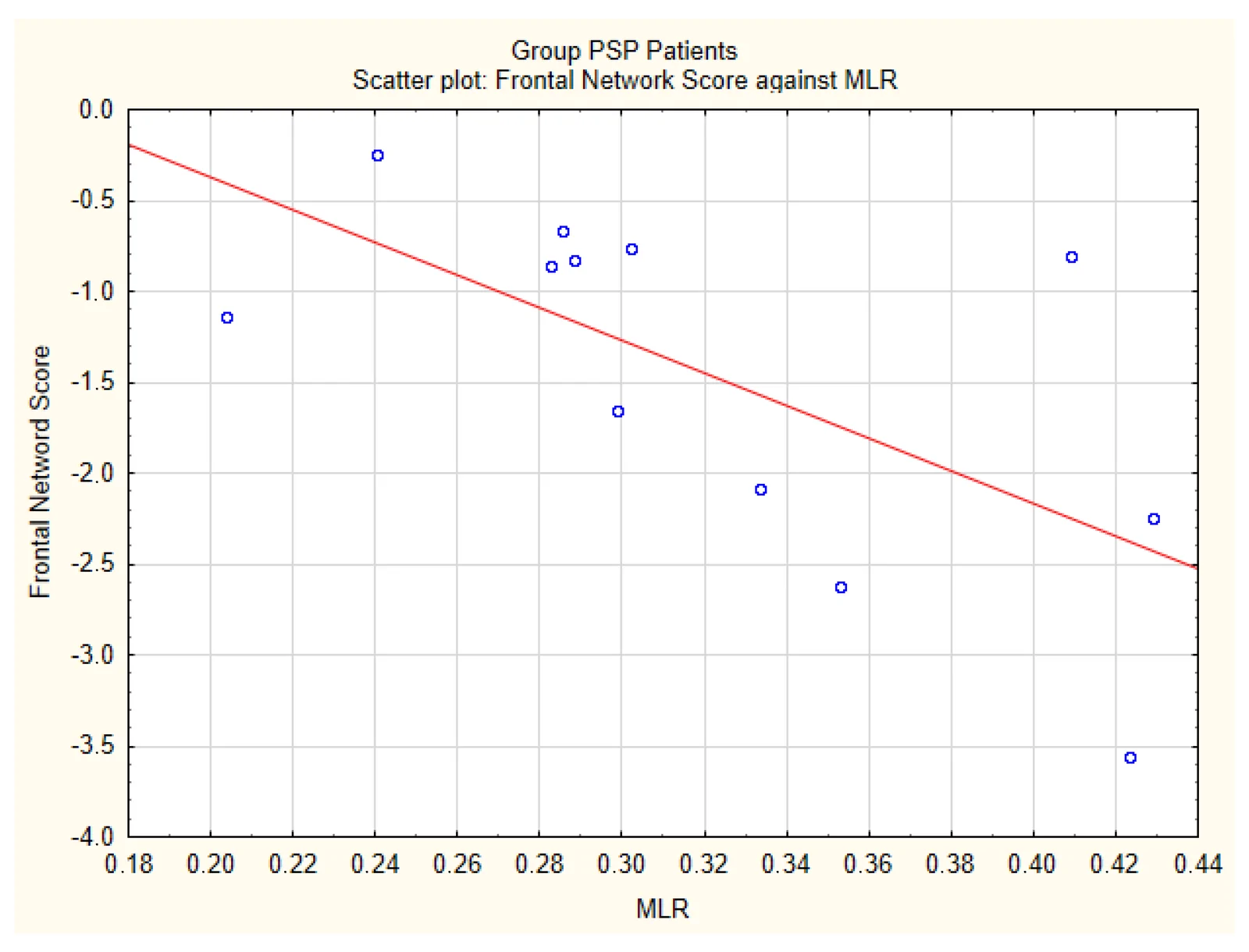
Association between the monocyte-to-lymphocyte ratio (MLR) and the composite Frontal Network Score in patients with progressive supranuclear palsy (PSP). Each point represents one participant. The red line illustrates the linear trend. Higher MLR values were associated with lower Frontal Network Score values (Spearman’s rho = −0.587, *p* = 0.045). After adjustment for age, this association remained significant after false discovery rate correction (partial r = −0.733, *p* = 0.0067, FDR q = 0.020).

To assess frontal network involvement at the systems level, a composite Frontal Network Score was calculated from normalized frontal lobe, MFC, SFG, and ACgG volumes. In unadjusted analyses, the strongest association was observed for MLR (rho = −0.587, *p* = 0.045). After adjustment for age, MLR remained significantly associated with lower Frontal Network Score values (partial r = −0.733, *p* = 0.0067, FDR q = 0.020), representing the only association that remained significant after correction for multiple comparisons. No significant associations were observed for NLR or SIRI after FDR correction (Figure 4, Table 5).

## 4. Discussion

The principal finding of the present study was the association between peripheral inflammatory burden and frontal network degeneration in progressive supranuclear palsy. Although several inflammatory indices showed inverse relationships with regional frontal volumes, monocyte-to-lymphocyte ratio (MLR) had the most robust association, remaining significantly related to the composite Frontal Network Score after adjustment for age and correction for multiple comparisons. In parallel, patients with PSP showed lower superior frontal gyrus and pallidal volumes than patients with Parkinson’s disease. This pattern supports the view that peripheral immune dysregulation may be linked to network-specific neurodegeneration affecting frontal executive circuits in PSP, a concept consistent with contemporary network and neuroinflammatory models of the disease [6,7,8,12,23,24,25].

The volumetric findings fit well with the established neuroanatomical profile of PSP. Neuropathological and neuroimaging studies have shown preferential involvement of the brainstem, pallidum, caudate nucleus, thalamus and frontal cortical regions, reflecting the selective vulnerability of fronto-striato-thalamo-cortical circuits to 4-repeat tau pathology [21,22,25]. In our cohort, superior frontal gyrus and pallidal volumes remained significantly reduced after correction for multiple testing. These results are in line with earlier morphometric data showing neostriatal and mesencephalic atrophy in PSP, as well as more recent longitudinal evidence indicating progressive involvement of subcortical and frontal regions across PSP clinical variants [21,22]. The pallidal finding is especially relevant, given the role of pallido-thalamo-cortical pathways in motor control, postural instability and executive dysfunction in PSP [21,22,25].

Recent network-based studies have changed the way PSP-related atrophy is interpreted. Rather than being a set of isolated regional changes, PSP increasingly appears to involve degeneration spreading along vulnerable functional and anatomical networks. Spinelli et al. showed that the spatial distribution of atrophy in PSP-RS follows the hierarchical organization of functional connectivity originating from the midbrain tegmentum, identified as a disease epicenter [24]. Functional connectivity abnormalities across PSP variants have also been described within fronto-subcortical, basal ganglia and thalamic circuits [23]. Against this background, the regional pattern observed in the present study is biologically plausible: inflammatory indices were related mainly to medial frontal cortex and superior frontal gyrus volumes, not uniformly to all analyzed regions.

A relevant negative finding should also be emphasized. None of the analyzed inflammatory indices differed significantly between PSP and PD. NLR, SIRI, and RDW-CV were numerically higher in PSP, whereas MLR was slightly lower. The direction of the NLR difference is consistent with previous reports of altered peripheral immune profiles in PSP. Muñoz-Delgado et al. found higher NLR values in both PSP and PD compared with healthy controls and showed that the peripheral immune profile in PSP was predominantly associated with higher neutrophil counts [12]. Their meta-analysis supported higher NLR and neutrophil counts in PSP compared with healthy controls, although substantial heterogeneity was present across studies [12]. Earlier studies have likewise suggested alterations in NLR in PSP and related atypical parkinsonian syndromes [10,11]. In our cohort, however, none of the between-group differences reached statistical significance, and the numerical differences should not be interpreted as evidence of greater systemic inflammatory activity in PSP. Instead, variability within the PSP cohort appeared to carry information about the degree of frontal network degeneration. This may partially explain why peripheral inflammatory markers have shown limited diagnostic discrimination despite increasing evidence of immune involvement in PSP.

The strongest regional association was observed between SIRI and medial frontal cortex volume. SIRI combines neutrophil, monocyte and lymphocyte counts and has been proposed as a broader marker of systemic inflammatory activation than NLR alone. In Parkinson’s disease, SIRI and related composite inflammatory indices have been associated with non-motor symptoms and clinical severity, although these observations remain disease-context dependent [13,15,17]. Data in PSP are still limited. In the present study, the inverse association between SIRI and medial frontal cortex volume supports the hypothesis that systemic inflammatory activity may parallel structural degeneration within vulnerable frontal networks. However, this result did not remain significant after FDR correction and should therefore be treated as a biologically coherent signal rather than a definitive independent finding.

The most compelling result involved MLR. Unlike SIRI, MLR remained significantly associated with the composite Frontal Network Score after age adjustment and FDR correction. This is important because the Frontal Network Score summarizes several interconnected frontal regions and may better capture system-level degeneration than isolated regional volumes. The persistence of the MLR association after statistical adjustment suggests that it is unlikely to be explained only by age or by variation in a single cortical measurement.

The inflammatory indices examined in this study should not be considered interchangeable. Although NLR, MLR and SIRI are all derived from peripheral blood cell counts, they capture partly different components of the circulating immune response. NLR reflects the relative contribution of neutrophils and lymphocytes and MLR places greater emphasis on the monocyte compartment, whereas SIRI incorporates neutrophil, monocyte and lymphocyte counts within a single index [12,13,14,15,16,17]. RDW-CV differs from the leukocyte-derived indices because it reflects variability in erythrocyte size rather than the relative abundance of circulating immune-cell populations. These differences may partly account for the non-uniform associations with MRI measures observed in our cohort. The association involving MLR is of particular interest in view of the involvement of monocyte–macrophage and microglial pathways in neurodegenerative inflammation [4,6,7,8,9]. However, given the small PSP sample, differences between individual correlation coefficients may also reflect sampling variability. Accordingly, only the age-adjusted association between MLR and the composite Frontal Network Score that survived FDR correction should be regarded as statistically robust in the present dataset.

The biological interpretation of MLR is plausible in the context of the current PSP literature. Monocytes and monocyte-derived mediators may influence microglial activation, cytokine signaling, endothelial function and blood–brain barrier integrity [6,7,8,9]. In PSP and other 4-repeat tauopathies, microglial activation is not merely a histological observation but an in vivo measurable process related to tau pathology and disease progression [6,7,8,21]. From this perspective, MLR may be interpreted as an exploratory peripheral signal of immune imbalance associated with central neurodegeneration [6,7,8,12]. This does not mean that MLR directly measures microglial activity; rather, it may capture a peripheral component of the broader immune state associated with frontal network damage.

Several recent studies support this interpretation. Malpetti et al. showed that neuroinflammation measured with PET predicts subsequent clinical progression in PSP, whereas MRI volumes alone did not show the same prognostic value in that cohort [6]. In a later study of primary 4-repeat tauopathies, regional microglial activation spatially paralleled tau PET patterns across functionally connected brain regions [8]. Post-mortem validation has further strengthened the interpretation of TSPO PET in PSP by showing that ante-mortem TSPO binding correlates with post-mortem CD68-positive phagocytic microglia and microglial TSPO expression [7]. These observations provide a strong mechanistic framework for linking immune activity, tau pathology and network degeneration.

The relationship between peripheral immune alterations and neurodegeneration is not restricted to PSP or PD. Alzheimer’s disease provides the most extensively studied example: circulating inflammatory mediators are altered in patients with AD [18], while molecular imaging studies have demonstrated regional associations between neuroinflammation and tau pathology [19]. More broadly, systemic inflammatory markers have been associated with structural brain measures, including cortical thickness and grey matter volume, although these relationships vary according to the inflammatory marker, population, and disease stage [28]. Similar immune mechanisms have been implicated across tauopathies, but evidence directly linking peripheral blood indices to regional brain atrophy remains limited. Peripheral inflammatory indices should therefore be regarded as indirect markers of a broader immune state accompanying neurodegeneration rather than direct measures of central inflammatory activity or neuronal loss.

The immune component of PSP is probably broader than microglial activation alone. Recent neuropathological evidence indicates increased cytotoxic T-cell infiltration within the midbrain in PSP, suggesting that adaptive immune responses may also participate in disease pathology [9]. This point matters for the present study because blood-derived indices such as MLR, NLR and SIRI are mixed, indirect markers. They do not isolate a single immune pathway, but they may reflect the balance between innate and adaptive immune compartments. The current results therefore fit better with a model of immune–network interaction than with a simple one-directional pathway from systemic inflammation to focal atrophy.

A cautious interpretation is still necessary. Peripheral inflammatory indices are nonspecific and may be influenced by aging, vascular comorbidity, metabolic disorders, medication use or subclinical systemic inflammation. Studies in Parkinson’s disease have shown that NLR, lymphocyte count and related inflammatory indices can be associated with clinical severity, motor subtype and CSF markers of neurodegeneration [13,14,15,16,29]. Longitudinal immune profiling has also demonstrated that peripheral immune shifts occur early in PD and are not unique to PSP [30]. These findings provide an important counterargument: the associations observed in our cohort should not be interpreted as PSP-specific blood biomarkers of inflammatory or neurodegenerative activity. They more likely represent a broader biological link between peripheral immune status and neurodegenerative burden.

Previous studies of inflammatory blood markers in atypical parkinsonian syndromes have focused mainly on diagnostic discrimination. Elevated NLR has been reported in PSP, and case–control and meta-analytic data suggest that PSP and PD both show increased peripheral inflammation compared with healthy controls, although the immune profile may differ between the two diseases [10,12]. Earlier studies also examined NLR and PLR across atypical parkinsonian syndromes, including PSP/CBS and PD/MSA-P comparisons, with an emphasis on group separation rather than structural correlates of neurodegeneration [10,31]. More recent work has explored inflammatory markers in relation to specific PSP clinical features, including depressive symptoms, supporting the idea that blood-based immune indices may reflect disease heterogeneity rather than a uniform diagnostic signal [32]. The present study adds a different perspective by linking these indices to MRI-derived measures of regional and network-level atrophy.

The regional specificity of the observed associations deserves attention. The strongest relationships clustered within frontal cortical structures, especially medial frontal cortex and superior frontal gyrus. Comparable associations were not consistently seen across all subcortical regions. This pattern argues against a simple relationship between systemic inflammation and global brain atrophy. Instead, it suggests that inflammatory activity may be most relevant to frontal executive networks, which are among the systems most consistently affected in PSP and closely connected with the subcortical disease epicenters described in contemporary network models [21,23,24,25].

## 5. Limitations

The present study has several limitations. The sample size was small, reflecting the rarity of PSP and limiting statistical power, particularly after correction for multiple comparisons. The relatively large number of correlation analyses in relation to sample size increased the risk of both type I and type II statistical errors despite application of FDR correction. The cross-sectional design does not allow causal inference: peripheral immune activation may contribute to neurodegeneration, occur secondary to neuronal injury, or develop in parallel with other disease mechanisms. Direct markers of neuroinflammation, such as TSPO PET, CSF cytokine profiling or neuropathological validation, were not available. Blood-derived indices also provide only indirect information and cannot distinguish monocyte subsets, lymphocyte phenotypes or cytokine pathways. An additional limitation is the absence of consistently available standardized measures of disease severity temporally matched to MRI and blood sampling. Regional brain volumes in PD may vary according to motor severity, including UPDRS Part III scores [33], and analogous effects of disease stage are relevant in PSP. Consequently, differences in disease severity between groups may have contributed to the observed volumetric differences. Stratification was not considered statistically appropriate given the small number of participants in each group. Future studies should prospectively acquire disease-specific severity measures and incorporate them as covariates in imaging analyses. Reliable retrospective assignment of MDS-defined PSP phenotypes was not possible for all participants because sufficiently detailed phenotypic information was not consistently available in the medical records. Consequently, potential differences in imaging or hematological associations across PSP clinical phenotypes could not be assessed. The study did not include an additional tauopathy comparison group. Comparison with AD would address a different clinical and neuroanatomical context, whereas inclusion of pathologically or biomarker-supported CBD would be particularly informative for determining whether the observed immune structural associations are specific to PSP or represent a feature shared across 4-repeat tauopathies. The absence of such a comparator limits the disease-specific interpretation of the present findings and should be addressed in future comparative studies. The study also lacked a healthy control group with corresponding MRI and hematological data. Consequently, the present design cannot determine whether the observed structural or blood-derived immune patterns represent abnormalities relative to healthy aging or primarily differences within the parkinsonian disease spectrum. Future studies incorporating PSP, PD, and matched healthy controls would provide a more comprehensive framework for assessing disease specificity. Finally, the composite Frontal Network Score was developed as an exploratory, biologically motivated measure of network-level degeneration and requires external validation in independent PSP cohorts.

## 6. Conclusions

PSP was associated with selective frontal and pallidal atrophy relative to PD. Within the PSP group, higher values of selected blood-derived inflammatory indices were associated with lower frontal network volumes, with MLR showing the most consistent association after adjustment for age and correction for multiple comparisons. These exploratory findings indicate an association between peripheral hematological immune-related measures and frontal network degeneration in PSP but do not establish a direct relationship between systemic inflammatory activity and neurodegeneration. Validation in larger longitudinal cohorts incorporating standardized clinical severity measures, molecular imaging, fluid inflammatory markers, and detailed immune profiling is required.

## Figures and Tables

**Table 1:** Demographic and blood-derived hematological characteristics of patients with progressive supranuclear palsy and Parkinson’s disease.

Variable	PD n = 12	PSP n = 12	*p*
Age	72.0 (67.0–73.0)	72.0 (68.2–74.0)	0.664
Male sex, n (%)	9 (75.0%)	7 (58.3%)	0.667
NLR	1.96 (1.74–2.34)	2.55 (2.03–3.04)	0.078
MLR	0.35 (0.27–0.37)	0.30 (0.28–0.37)	0.624
SIRI	1.31 (0.92–1.42)	1.39 (0.99–1.87)	0.237
RDW-CV	12.3 (12.1–13.0)	13.0 (12.4–13.4)	0.235

**Abbreviations:** MLR, monocyte-to-lymphocyte ratio; NLR, neutrophil-to-lymphocyte ratio; PD, Parkinson’s disease; PSP, progressive supranuclear palsy; RDW-CV, red blood cell distribution width coefficient of variation; SIRI, systemic inflammation response index. **Note:** Continuous variables are presented as median (interquartile range, IQR), whereas categorical variables are presented as number (percentage). Group comparisons were performed using the Mann–Whitney U test for continuous variables and Fisher’s exact test for categorical variables.

**Table 2:** Comparison of predefined MRI volumetric measures between patients with Parkinson’s disease and progressive supranuclear palsy.

MRI Variable	PD Median (IQR)	PSP Median (IQR)	*p*	FDR q
SFG total volume %	2.003 (1.955–2.062)	1.766 (1.710–1.866)	0.00048	0.0043
Pallidum total volume %	0.190 (0.170–0.203)	0.151 (0.113–0.160)	0.00165	0.0074
Frontal total volume %	11.424 (11.256–11.810)	11.070 (10.698–11.285)	0.035	0.091
Caudate total volume %	0.475 (0.464–0.522)	0.440 (0.407–0.481)	0.040	0.091
Brainstem volume %	1.350 (1.248–1.492)	1.239 (1.171–1.320)	0.061	0.109
ACgG total volume %	0.688 (0.663–0.719)	0.640 (0.566–0.683)	0.100	0.144
MFC total volume %	0.212 (0.203–0.219)	0.192 (0.173–0.218)	0.141	0.158
Thalamus total volume %	0.734 (0.684–0.765)	0.687 (0.620–0.733)	0.285	0.285

**Abbreviations:** ACgG, anterior cingulate gyrus; FDR, false discovery rate; IQR, interquartile range; MFC, medial frontal cortex; PD, Parkinson’s disease; PSP, progressive supranuclear palsy; SFG, superior frontal gyrus. **Note:** Data are presented as median (IQR). All volumetric measurements are expressed as percentages of intracranial volume. *p*-values were obtained using the Mann–Whitney U test. False discovery rate correction was performed using the Benjamini–Hochberg method.

**Table 3:** Associations between peripheral inflammatory markers and frontal network volumetric measures in patients with progressive supranuclear palsy.

Marker	MRI Variable	Spearman Rho	*p*	FDR q
SIRI	MFC total volume %	−0.748	0.0051	0.061
NLR	SFG total volume %	−0.650	0.022	0.092
NLR	MFC total volume %	−0.643	0.024	0.092
MLR	MFC total volume %	−0.622	0.031	0.092
MLR	SFG total volume %	−0.601	0.039	0.093
NLR	Frontal total volume %	−0.538	0.071	0.142

**Abbreviations:** FDR, false discovery rate; MFC, medial frontal cortex; MLR, monocyte-to-lymphocyte ratio; NLR, neutrophil-to-lymphocyte ratio; PSP, progressive supranuclear palsy; SFG, superior frontal gyrus; SIRI, systemic inflammation response index. **Note:** Correlations were assessed using Spearman’s rank correlation coefficient (rho). All volumetric measurements are expressed as percentages of intracranial volume. The analysis was restricted to predefined frontal network regions selected *a priori* based on current neuropathological models of PSP. *p*-values were adjusted for multiple comparisons using the Benjamini–Hochberg false discovery rate procedure. Negative correlation coefficients indicate that higher levels of peripheral inflammatory markers were associated with lower normalized frontal network volumes.

**Table 4:** Age-adjusted associations between peripheral inflammatory markers and frontal network volumetric measures in patients with progressive supranuclear palsy.

Marker	MRI Variable	Partial r	*p*	FDR q
SIRI	MFC total volume %	−0.734	0.0066	0.099
NLR	MFC total volume %	−0.621	0.031	0.112
MLR	MFC total volume %	−0.612	0.034	0.112
NLR	SFG total volume %	−0.604	0.037	0.112

**Abbreviations:** FDR, false discovery rate; MFC, medial frontal cortex; MLR, monocyte-to-lymphocyte ratio; NLR, neutrophil-to-lymphocyte ratio; SFG, superior frontal gyrus; SIRI, systemic inflammation response index. **Note:** Partial correlation coefficients were adjusted for age. *p*-values were corrected for multiple comparisons using the Benjamini–Hochberg false discovery rate procedure. Negative correlation coefficients indicate an association between higher inflammatory marker values and lower normalized regional brain volumes.

**Table 5:** Associations between peripheral inflammatory markers and the composite Frontal Network Score in progressive supranuclear palsy.

Inflammatory Marker	Spearman Rho	*p*-Value	FDR q-Value	Age-Adjusted Partial r	*p*-Value	FDR q-Value
NLR	−0.462	0.131	0.196	−0.596	0.041	0.061
MLR	−0.587	0.045	0.134	−0.733	0.0067	0.020
SIRI	−0.266	0.404	0.404	−0.487	0.108	0.108

**Abbreviations:** FDR, false discovery rate; MLR, monocyte-to-lymphocyte ratio; NLR, neutrophil-to-lymphocyte ratio; SIRI, systemic inflammation response index. **Note:** The composite Frontal Network Score was calculated as the mean z-score of normalized frontal lobe, medial frontal cortex, superior frontal gyrus, and anterior cingulate gyrus volumes. All MRI volumetric parameters were expressed as percentages of intracranial volume. Age-adjusted associations were assessed using partial correlation coefficients. *p*-values were adjusted using the Benjamini–Hochberg false discovery rate procedure. Lower Frontal Network Score values indicate lower normalized frontal network volumes.

## Data Availability

The data that support the findings of this study are not publicly available because they contain potentially identifiable participant information. De-identified data may be available from the corresponding author on request, subject to ethics approval and institutional requirements.
